# Old-age-induced obesity reversed by a methionine-deficient diet or oral administration of recombinant methioninase-producing *Escherichia coli* in C57BL/6 mice

**DOI:** 10.18632/aging.204783

**Published:** 2023-06-09

**Authors:** Yutaro Kubota, Qinghong Han, Jose Reynoso, Yusuke Aoki, Noriyuki Masaki, Koya Obara, Kazuyuki Hamada, Michael Bouvet, Takuya Tsunoda, Robert M. Hoffman

**Affiliations:** 1 AntiCancer Inc., San Diego, CA 92111, USA; 2 Department of Surgery, University of California, San Diego, CA 92111, USA; 3 Department of Medical Oncology, Division of Internal Medicine, Showa University School of Medicine, Tokyo, Japan

**Keywords:** obesity, aging, methionine restriction, methionine-deficient diet, recombinant methioninase (rMETase), *Escherichia coli*, microbiome, weight-loss

## Abstract

Obesity increases with aging. Methionine restriction affects lipid metabolism and can prevent obesity in mice. In the present study we observed C57BL/6 mice to double their body weight from 4 to 48 weeks of age and become obese. We evaluated the efficacy of oral administration of recombinant-methioninase (rMETase)-producing *E. coli* (*E. coli* JM109-rMETase) or a methionine-deficient diet to reverse old-age-induced obesity in C57BL/6 mice. Fifteen C57BL/6 male mice aged 12–18 months with old-age-induced obesity were divided into three groups. Group 1 was given a normal diet supplemented with non-recombinant *E. coli* JM109 cells orally by gavage twice daily; Group 2 was given a normal diet supplemented with recombinant *E. coli* JM109-rMETase cells by gavage twice daily; and Group 3 was given a methionine-deficient diet without treatment. The administration of *E. coli* JM109-rMETase or a methionine-deficient diet reduced the blood methionine level and reversed old-age-induced obesity with significant weight loss by 14 days. There was a negative correlation between methionine levels and negative body weight change. Although the degree of efficacy was higher in the methionine-deficient diet group than in the *E. coli* JM109-rMETase group, the present findings suggested that oral administration of *E. coli* JM109-rMETase, as well as a methionine-deficient diet, are effective in reversing old-age-induced obesity. In conclusion, the present study provides evidence that restricting methionine by either a low-methionine diet or *E. coli* JM109-rMETase has clinical potential to treat old-age-induced obesity.

## INTRODUCTION

Globally, the incidence of obesity is increasing [1, 2]. In general, people tend to become obese when they get older due to being hypokinetic and having decreased rates of metabolism [3]. Population-based studies have identified obesity as a risk factor for an increasing number of chronic diseases, such as cardiovascular disease, diabetes mellitus, chronic renal disease [4], several malignancies [5], and various musculoskeletal disorders [6]. However, once people get become obese, it becomes difficult to lose weight [7, 8].

Methionine is an amino acid and plays an essential role in our body. Methionine is the N-terminal amino acid in nuclear-encoded proteins, and its metabolite S-adenosyl methionine (SAM) is the cell’s main methyl group provider [9]. Methionine restriction was effective to prevent body weight gain in rodents which were fed a methionine-restricted diet starting early in life [10, 11]. Methionine restriction enhances de novo lipogenesis, lipolysis, and fatty acid oxidation, resulting in decreased in fat formation. [12]. In the present study we tested a low-methionine diet to reverse old-age-induced obesity.

All protein sources include methionine, making it impossible to strictly limit methionine by diet alone. Therefore, we have developed recombinant-methioninase (rMETase), an enzyme that degrades methionine [13–15]. We previously reported that oral rMETase prevents obesity in mice fed a high-fat diet [16]. We also reported that oral administration of rMETase-producing *E. coli* JM109 (*E. coli* JM109-rMETase) inhibited tumor growth *in vivo* [17]. Therefore *E. coli* JM109-rMETase was also tested in the present study to reverse old-age-induced obesity.

## RESULTS

### Increase of mouse body weight with age

**Figure 1:**
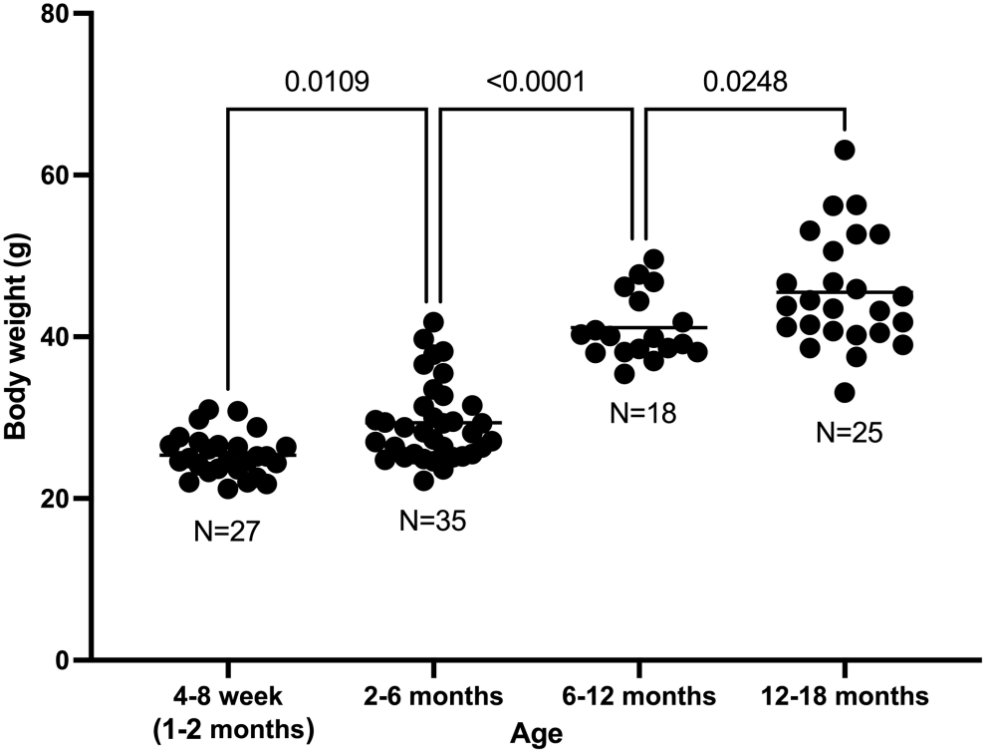
Body weight increase with age in C57BL/6 male mice.

Mouse body weight increased with age. The mean body weight in mice aged 1, 2 months was 25.4 g; 2–6 months was 29.4 g (*p* = 0.0109 with respect to 1–2 months); 6–12 months was 41.1 g (*p* < 0.0001 with respect to 2–6 months); and 12–18 months was 45.5 g (*p* = 0.0248 with respect to 6–12 months). (Figure 1).

### Obesity was reversed by oral administration. rMETase-producing *E. coli* JM109

**Figure 2:**
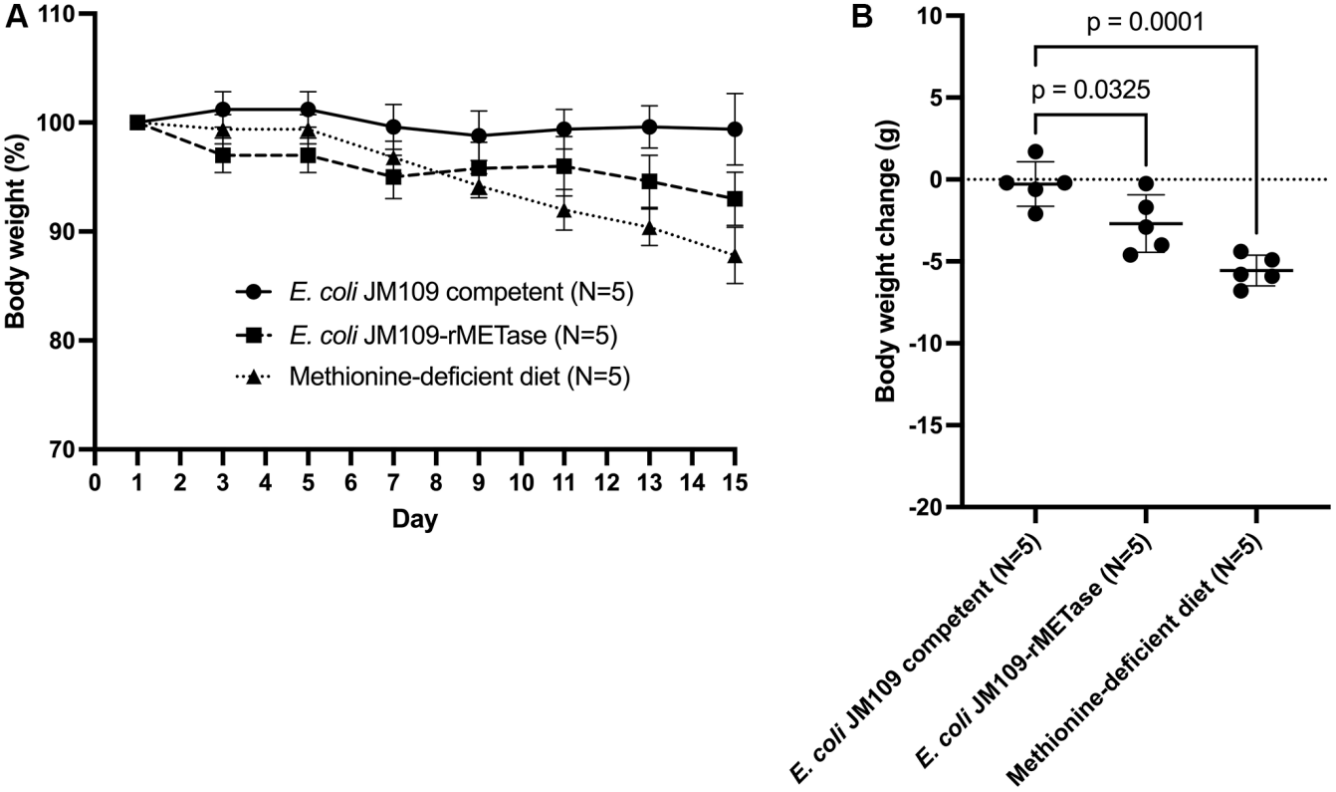
(**A**) Percent body weight change from study days 1–15. (**B**) Body weight change from baseline in each group on day 15.

Figure 2A shows each group’s percent body weight change for 14 days. In mice fed non-recombinant *E. coli* JM109 cells (control group), mouse body weight did not change significantly during 14 days. The mice administered recombinant *E. coli* JM109-rMETase (*E. coli* JM109-rMETase group) decreased their mean body weight from 43.8 g to 40.7 g by day 15. This 3.1 g body weight loss is significantly higher than that of the control group (*p* = 0.0325). In the mice fed a methionine- and choline-deficient diet (methionine-deficient diet group), body-weight loss was 5.6 g (*p* = 0.0001). Mouse body weight continued to decrease until 15 days on the low-methionine diet. (Figure 2B).

**Figure 3:**
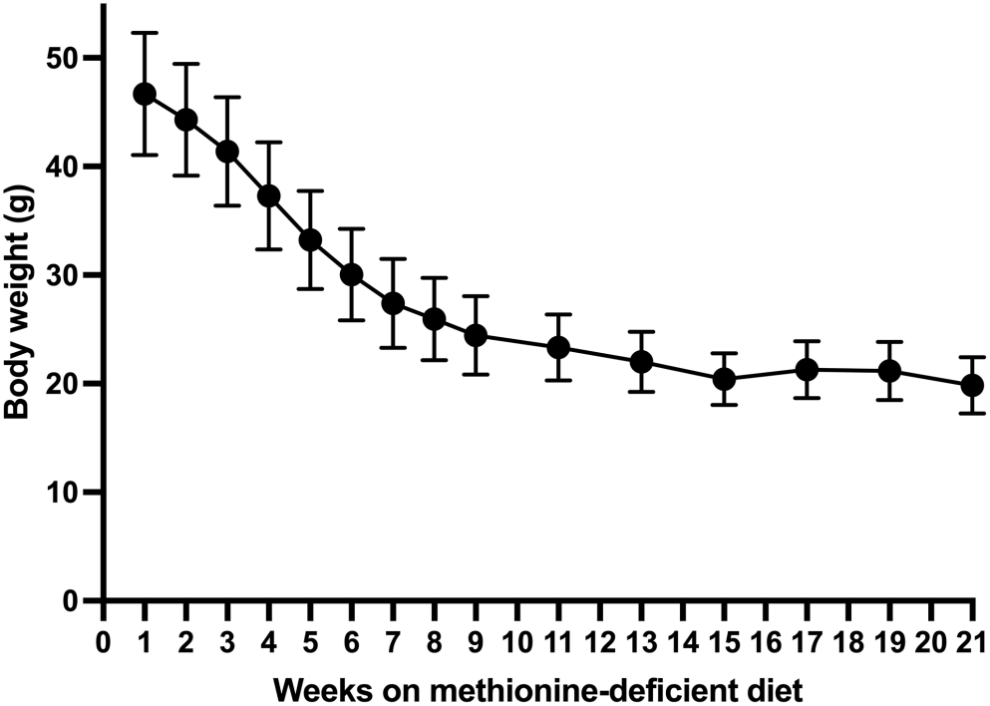
Long-term body weight change in mice on the methionine-deficient diet.

After day 15, the body weight of the methionine-deficient-diet group continued to decrease until week 15 with a total average loss of 26.3 g. (Figure 3) Fifteen weeks after the beginning of the methionine-deficient diet, mouse body weight stabilized at 20 grams.

### Methionine level

**Figure 4:**
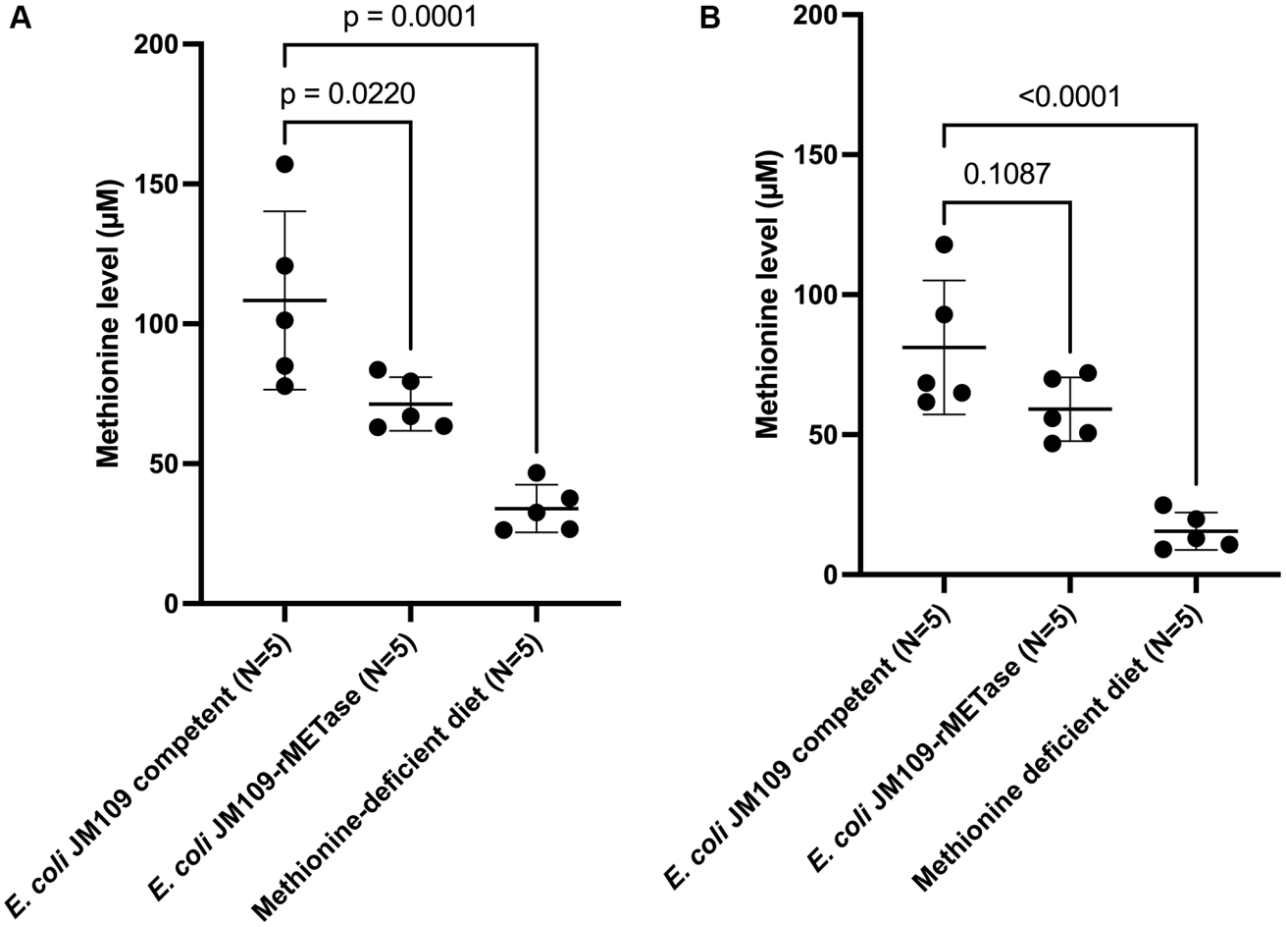
Blood methionine level at day 15 (**A**) and day 29 (**B**).

**Figure 5:**
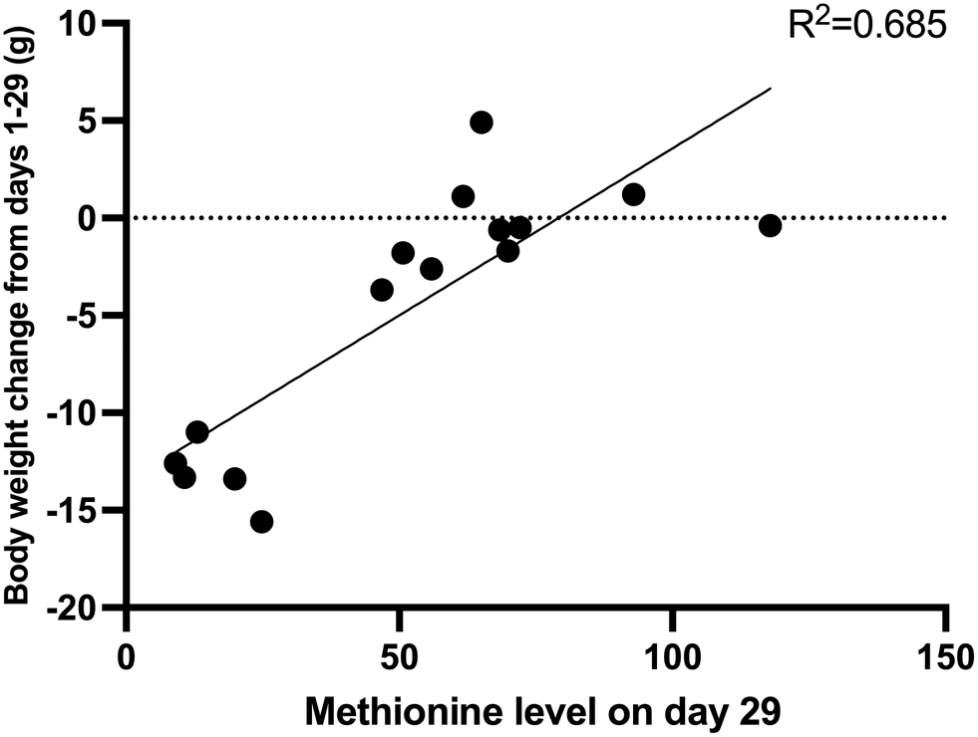
The correlation between methionine level and body weight change on day 29.

On day 15, the mean methionine level of each group was as follows: control group: 108.4 μM; *E. coli* JM109-rMETase group: 71.3 μM; methionine-deficient diet group: 34.0 μM. Both the methionine-deficient diet group and the *E. coli* JM109-rMETase-treated group showed significantly lower levels of methionine compared to the control group (Figure 4A). On day 29, the mean methionine level of each group was 81.2, 59.1, and 15.5 μM, respectively (Figure 4B). There was a negative correlation between the methionine level and negative body-weight change on day 29, R^2^ = 0.685, (Figure 5).

### Stool culture of *E. coli* JM109-rMETase group

*E. coli* JM109-rMETase was detected in the *E. coli* JM109-rMETase group stool on day 15 and day 22 indicating incorporation in the microbiome.

## DISCUSSION

This is the first report that showed the efficacy of methionine restriction to reverse old-age-induced obesity. The correlation between age and body weight shown in the present study is consistent with previous data [11]. Similar to humans, mice become obese as they get older. Both *E. coli* JM109-rMETase and a methionine deficient diet lowered methionine levels and reversed obesity. The degree of efficacy of lowering methionine and reversal of obesity was superior in the methionine-deficient diet group compared to treatment with *E. coli* JM109-rMETase. It is notable that the body weight loss of the mice on the low methionine diet stabilized at week 15 at 20 grams. Thus, the low-methionine seems to have decreased adipose tissue and not lean muscle mass.

Different from mice, it is difficult for human beings to restrict methionine strictly only by diet. Therefore, we have developed oral methioninase and *E. coli* JM109-rMETase. Recently an engineered *E. coli* Nissle, designed to metabolize methionine via the methionine decarboxylase pathway, showed about 25% blood methionine level reduction for healthy volunteers in a phase I study [18]. Although this probiotic was developed for the treatment of homocystinuria, these results showed that this type of probiotic therapy, similar to our *E. coli* JM109-rMETase, can decrease the blood methionine level in human patients.

In conclusion, methionine restriction using oral installation of recombinant methioninase-producing *Escherichia coli* or a methionine-deficient diet is effective to reverse old-age-induced obesity, both of which have clinical potential.

## MATERIALS AND METHODS

### Mice

C57BL/6 male mice (AntiCancer Inc., San Diego, CA, USA) aged 1-18 months were used in the present study. The mice were housed in a barrier facility with a HEPA-filtered rack under typical light/dark cycles of 12 hours. Mice were given an autoclaved laboratory rodent meal before the beginning of this study. The AntiCancer Institutional Animal Care and Use Committee’s ethics committee granted approval in accordance with National Institutes of Health Guide Assurance Number 3873-1. All experiments adhered to the Animal Research: Reporting of *In Vivo* Experiments (ARRIVE) 2.0 guidelines [19].

### Culture of *E. coli* JM109-rMETase

The host strain for the expression of rMETase was *E. coli* JM109. The *P. putida* rMETase gene was previously cloned into *E. coli* JM109 using the plasmid pATG3131, which also contains the tetracycline (TC) resistance gene [14, 15]. The generated *E. coli* JM109-rMETase was pre-cultured in 5 ml of liquid Luria-Bertani (LB) medium with TC (32 g/ml) for eight hours at 37°C. The pre-culture broth was transferred overnight to 400 ml culture medium containing 32 g/ml TC. To promote the expression of rMETase, isopropyl—D-thiogalactopyranoside (IPTG) was administered at a final concentration of 0.3 mM for 4 hours at 28°C. The concentration of *E. coli* JM109-rMETase was adjusted to 10^11^ CFU/mL using phosphate-buffered saline (PBS) and 20% glycerin. *E. coli* JM109-rMETase was stored at −80°C until administration to mice. Non-recombinant *E. coli* JM109 competent cells were generated in the same manner as *E. coli* JM109-rMETase, except for the addition of TC and IPTG to the LB medium.

### Study design

Spontaneous body-weight gain was observed in C57BL/6 mice for up to 18 months. Fifteen mice weighing more than 40 g and aged 12 to 18 months were chosen and divided into three groups of five mice each, with similar mean weight in each group: Group 1: normal diet gavaged with non-recombinant *E. coli* JM109 competent cells (10^10^/100 μL), treated twice daily (9 AM and 5 PM by oral gavage) for 14 days: Group 2: Normal diet supplemented with *E. coli* JM109-rMETase cells (10^10^/100 μL), treated by gavage twice daily (9 AM and 5 PM) for 14 days. Group 3: Mice on a methionine-deficient and choline-diet without treatment for 21 weeks. The normal diet contained 0.5% methionine, while the methionine-deficient diet was depleted of methionine, homocysteine, and choline.

IPTG (10 mM) and TC (0.5 g/L) were added to the drinking water of the mice to induce *E. coli* JM109-rMETase to produce rMETase and prevent plasmid shedding only in group 2 treated with *E. coli* JM109-rMETase [15]. Body weight was measured every two days until day 15, then every seven days until 21 weeks.

### Blood and stool collection

Mouse blood was obtained through tail hemorrhage at 9 to 10 o’clock in the morning on days 15 and 29. Only the mice that received *E. coli* JM109-rMETase had their stool collected in the mornings of days 15, 22, and 29.

### Mouse stool culture and screening for *E. coli* JM109-rMETase in the stool

The stool was diluted 1:10 by weight with PBS, and then mechanically disrupted. Large debris was pelletized by brief centrifugation at 200 × g. Subsequently, 100 μL of the supernatant was plated onto LB agar with 32 μg/mL TC and incubated at 37°C overnight. The screening of *E. coli* JM109-rMETase in the stool was performed using modified M9 agar as described previously [17].

### Determination of plasma methionine level

The plasma concentration of methionine was measured using precolumn derivatization, followed by high-performance liquid chromatography separation (HPLC) based on a previously described method [20].

### Statistical analysis

GraphPad Prism 9.4.0 was used to perform all statistical analyses (GraphPad Software, Inc., San Diego, CA, USA). Tukey-Kramer was used for the parametric test of group comparison. All data are represented by the mean and standard deviation. *p*-Values less than 0.05 were considered significant.

## References

[1] Flegal KM, Carroll MD, Ogden CL, Curtin LR. Prevalence and trends in obesity among US adults, 1999-2008. JAMA. 2010; 303:235–41. 10.1001/jama.2009.2014 20071471

[2] Afshin A, Forouzanfar MH, Reitsma MB, Sur P, Estep K, Lee A, Marczak L, Mokdad AH, Moradi-Lakeh M, Naghavi M, Salama JS, Vos T, Abate KH, et al, and GBD 2015 Obesity Collaborators. Health Effects of Overweight and Obesity in 195 Countries over 25 Years. N Engl J Med. 2017; 377:13–27. 10.1056/NEJMoa1614362 28604169 PMC5477817

[3] Roberts SB, Dallal GE. Energy requirements and aging. Public Health Nutr. 2005; 8:1028–36. 10.1079/phn2005794 16277818

[4] Lavie CJ, Milani RV, Ventura HO. Obesity and Cardiovascular Disease: Risk Factor, Paradox, and Impact of Weight Loss. J Am Coll Cardiol. American College of Cardiology Foundation. 2009; 53:1925–32. 10.1016/j.jacc.2008.12.068 19460605

[5] Calle EE, Kaaks R. Overweight, obesity and cancer: epidemiological evidence and proposed mechanisms. Nat Rev Cancer. 2004; 4:579–91. 10.1038/nrc1408 15286738

[6] Wearing SC, Hennig EM, Byrne NM, Steele JR, Hills AP. Musculoskeletal disorders associated with obesity: a biomechanical perspective. Obes Rev. 2006; 7:239–50. 10.1111/j.1467-789X.2006.00251.x 16866972

[7] Hill JO, Wyatt HR, Reed GW, Peters JC. Obesity and the environment: where do we go from here? Science. 2003; 299:853–5. 10.1126/science.1079857 12574618

[8] Proietto J. Why is treating obesity so difficult? Justification for the role of bariatric surgery. Med J Aust. 2011; 195:144–6. 10.5694/j.1326-5377.2011.tb03242.x 21806533

[9] Sanderson SM, Gao X, Dai Z, Locasale JW. Methionine metabolism in health and cancer: a nexus of diet and precision medicine. Nat Rev Cancer. 2019; 19:625–37. 10.1038/s41568-019-0187-8 31515518

[10] Orentreich N, Matias JR, DeFelice A, Zimmerman JA. Low methionine ingestion by rats extends life span. J Nutr. 1993; 123:269–74. 10.1093/jn/123.2.269 8429371

[11] Miller RA, Buehner G, Chang Y, Harper JM, Sigler R, Smith-Wheelock M. Methionine-deficient diet extends mouse lifespan, slows immune and lens aging, alters glucose, T4, IGF-I and insulin levels, and increases hepatocyte MIF levels and stress resistance. Aging Cell. 2005; 4:119–25. 10.1111/j.1474-9726.2005.00152.x 15924568 PMC7159399

[12] Zhou X, He L, Wan D, Yang H, Yao K, Wu G, Wu X, Yin Y. Methionine restriction on lipid metabolism and its possible mechanisms. Amino Acids. 2016; 48:1533–40. 10.1007/s00726-016-2247-7 27156065

[13] Hoffman RM. Development of recombinant methioninase to target the general cancer-specific metabolic defect of methionine dependence: a 40-year odyssey. Expert Opin Biol Ther. 2015; 15:21–31. 10.1517/14712598.2015.963050 25439528

[14] Tan Y, Xu M, Tan X, Tan X, Wang X, Saikawa Y, Nagahama T, Sun X, Lenz M, Hoffman RM. Overexpression and large-scale production of recombinant L-methionine-alpha-deamino-gamma-mercaptomethane-lyase for novel anticancer therapy. Protein Expr Purif. 1997; 9:233–45. 10.1006/prep.1996.0700 9056489

[15] Takakura T, Ito T, Yagi S, Notsu Y, Itakura T, Nakamura T, Inagaki K, Esaki N, Hoffman RM, Takimoto A. High-level expression and bulk crystallization of recombinant L-methionine gamma-lyase, an anticancer agent. Appl Microbiol Biotechnol. 2006; 70:183–92. 10.1007/s00253-005-0038-2 16012835

[16] Tashiro Y, Han Q, Tan Y, Sugisawa N, Yamamoto J, Nishino H, Inubushi S, Higuchi T, Aoki T, Murakami M, Hoffman RM. Oral Recombinant Methioninase Prevents Obesity in Mice on a High-fat Diet. In Vivo. 2020; 34:489–94. 10.21873/invivo.11799 32111745 PMC7157881

[17] Kubota Y, Han Q, Hamada K, Aoki Y, Masaki N, Obara K, Baranov A, Bouvet M, Tsunoda T, Hoffman RM. Oral Installation of Recombinant Methioninase-producing Escherichia coli into the Microbiome Inhibits Colon-cancer Growth in a Syngeneic Mouse Model. Cancer Genomics Proteomics. 2022; 19:683–91. 10.21873/cgp.20351 36316039 PMC9620449

[18] Synlogic Initiates Phase 1 Study of SYNB1353 for the Treatment of Homocystinuria (HCU). 2022; https://investor.synlogictx.com/news-releases/news-release-details/synlogic-initiates-phase-1-study-synb1353-treatment.

[19] Percie du Sert N, Ahluwalia A, Alam S, Avey MT, Baker M, Browne WJ, Clark A, Cuthill IC, Dirnagl U, Emerson M, Garner P, Holgate ST, Howells DW, et al. Reporting animal research: Explanation and elaboration for the ARRIVE guidelines 2.0. PLoS Biol. 2020; 18:e3000411. 10.1371/journal.pbio.3000411 32663221 PMC7360025

[20] Sun X, Tan Y, Yang Z, Li S, Hoffman RM. A rapid HPLC method for the measurement of ultra-low plasma methionine concentrations applicable to methionine depletion therapy. Anticancer Res. 2005; 25:59–62. 15816519

